# Retraction: Modulatory Role of PYY in Transport and Metabolism of Cholesterol in Intestinal Epithelial Cells

**DOI:** 10.1371/journal.pone.0267059

**Published:** 2022-04-14

**Authors:** The PLOS ONE Editors

Following the publication of this article [1], concerns were raised regarding results presented in Figs 2, 4, 8, 9, 12, 13, and 14. Specifically,

- The following results appear similar:
  ○ Fig 4 left β-actin panel lanes 3 and 5, Fig 4 right β-actin panel lanes 3 and 5, and Fig 4 bottom β-actin panel lanes 1, 2, and 3.
  ○ Fig 4 left β-actin panel lane 4, and Fig 4 right β-actin panel lanes 2 and 4.
  ○ Fig 8 ACAT-2 panel lanes 2 and 4.
  ○ Fig 9 β-actin panel lanes 1 and 5
  ○ Fig 13A RXRα panel lanes 2 and 5.
  ○ Figs 13A, 13B, 13C, 13D, 14B, and 14D GAPDH panels
  ○ Fig 13B RXRβ panel lanes 1 and 4.
  ○ Fig 13C LXRα panel lane 5 and Fig 13D LXRβ panel lane 1.
  ○ Fig 14A PPARα lanes 1–2 and Fig 14C PPARγ lanes 4–5.
  ○ Fig 14A and Fig 14C GAPDH panels.
  ○ Fig 14B PPARβ panel lanes 2 and 5.
  ○ Fig 14D SREBP2 panel lanes 1 and 4.
- When levels are adjusted to visualize background, there appear to be vertical irregularities suggestive of splice lines in:
  ○ Fig 2 NPY1R panel, between all lanes
  ○ Fig 4 NPC1L1 panel, between lanes 2–3 and lanes 3–4.
  ○ Fig 4 CD36 panel, between lanes 3–4.
  ○ Fig 12 MTP panel, between lanes 3–4.
  ○ Fig 13C LXRα panel, between lanes 4–5.
  ○ Fig 14C PPARγ panel, between lanes 2–3.
  ○ Fig 14D SREBP2 panel, between lanes 2–3.

The corresponding author states that the similarity between proteins is due to the stripping and re-probing of the same blot, and clarifies that some blots were spliced to remove extra lanes and/or duplicate or triplicate samples from the panel. The underlying data for the figures or concern are no longer available.

In light of the concerns affecting multiple figure panels that question the integrity of these data, the *PLOS ONE* Editors retract this article.

EG, CG and ED either did not respond directly or could not be reached. EL did not agree with the retraction.
